# Adapted Taekwondo Improves Postural Balance and Health-Related Quality of Life Concerning Multicomponent Training and Walking Exercise in Older Females: A Randomized Controlled Trial (TKD and Aging Project)

**DOI:** 10.3390/jcm13237250

**Published:** 2024-11-28

**Authors:** Pablo Valdés-Badilla, Tomás Herrera-Valenzuela, Eduardo Guzmán-Muñoz, Jordan Hernandez-Martinez, Izham Cid-Calfucura, Edgar Vásquez-Carrasco, Juan Aristegui-Mondaca, Pablo Aravena-Sagardia, Jorge Mota, José Zapata-Bastias, Cristian Luarte-Rocha, Braulio Henrique Magnani Branco

**Affiliations:** 1 Department of Physical Activity Sciences, Faculty of Education Sciences, Universidad Católica del Maule, Talca 3530000, Chile; valdesbadilla@gmail.com; 2 Sports Coach Career, School of Education, Universidad Viña del Mar, Viña del Mar 2520000, Chile; jzapata@uvm.cl; 3 Department of Physical Activity, Sports and Health Sciences, Faculty of Medical Sciences, Universidad de Santiago de Chile (USACH), Santiago 8370003, Chile; izham.cid@gmail.com; 4 School of Kinesiology, Faculty of Health, Universidad Santo Tomás, Talca 3460000, Chile; eguzmanm@santotomas.cl; 5 School of Kinesiology, Faculty of Health Sciences, Universidad Autónoma de Chile, Talca 3460000, Chile; 6 Department of Physical Activity Sciences, Universidad de Los Lagos, Osorno 5290000, Chile; jordan.hernandez@ulagos.cl; 7 G-IDyAF Research Group, Department of Physical Activity Sciences, Universidad de Los Lagos, Osorno 5290000, Chile; 8 Programa de Investigación en Deporte, Sociedad y Buen Vivir, Universidad de Los Lagos, Osorno 5290000, Chile; 9 Occupational Therapy School, Faculty of Psychology, University de Talca, Talca 3460000, Chile; edgar.vasquez@utalca.cl; 10 Physical Education School, Faculty of Education Sciences, Universidad Católica del Maule, Talca 3530000, Chile; juan.aristegui@alu.ucm.cl; 11 Physical Education Pedagogy, Faculty of Education, Universidad Autónoma de Chile, Temuco 4780000, Chile; pablo.aravena@uautonoma.cl; 12 Research Centre of Physical Activity, Health, and Leisure, Laboratory for Integrative and Translational Research in Population Health (ITR), Faculty of Sports, University of Porto, 4200-450 Porto, Portugal; jmota@fade.up.pt; 13 Facultad de Odontología y Ciencias de la Rehabilitación, Universidad de San Sebastían, Concepción 4080871, Chile; cristian.luarte@uss.cl; 14 Postgraduate Program in Health Promotion, Cesumar University, Maringá 87050-390, Paraná, Brazil; braulio.branco@unicesumar.edu.br

**Keywords:** physical activity, combat sports, resistance training, older adults, healthy aging, aging

## Abstract

**Background/Objectives:** This study aimed to assess and compare the effects of an adapted taekwondo (TKD) program, multicomponent training (MCT), walking exercise (WE), and inactive control group (CG) on blood pressure, morphological variables, frequency of food consumption, cognitive status, health-related quality of life (HRQoL), physical fitness tests, and postural balance in independent older females. **Methods:** A randomized controlled trial study was conducted with the following groups: TKD (n = 13), MCT (n = 12), WE (n = 12), and CG (n = 14), considering three/weekly 60-min/sessions for 16-weeks. A two-factor mixed analysis of the variance model with repeated measures was performed. **Results:** TKD improved significantly more in phonetic fluency (*p* = 0.021; ES = 1.89) than WE and in general health (*p* = 0.033; ES = 1.11) than CG. Both TKD and MCT improved significantly more than CG in the 30 s chair stand, arm curl, chair sit-and-reach, timed up-and-go, maximal isometric handgrip strength, and postural balance for the eyes closed condition in the area and anteroposterior velocity (*p <* 0.05). **Conclusions:** Only TKD improved the area (*p* = 0.008; ES = 1.00) and mediolateral velocity (*p* = 0.019; ES = 0.79) for the eyes open condition, and mediolateral velocity (*p* = 0.021; ES = 1.57) for the eyes closed condition. Blood pressure, morphological variables, and food consumption frequency showed no significant intragroup or intergroup interactions. TKD equivalently improved HRQoL and physical fitness to MCT, with better postural balance in older females.

## 1. Introduction

Olympic combat sports (OCSs) are a novel alternative to improve functional performance in older people [1]. Several systematic reviews have reported positive benefits with OCS interventions on balance, fall risk [1], cardiorespiratory fitness [2], cognitive function [3], and health-related quality of life (HRQoL) [4]. Among the OCSs analyzed, karate, boxing, judo, and taekwondo are the most commonly used for interventions with older people [1,3,4].

Specifically, adapted taekwondo (TKD) has been used as a physical activity intervention in several randomized controlled trials with older females. For example, Cho and Roh [5], in a TKD intervention, showed better performance in the 2-min step, 30 s chair stand, and sit-and-reach tests compared to an inactive control group (CG). Similarly, a study carried out by Baek, et al. [6] in older females with depression and dementia reported a significant increase in maximal isometric handgrip strength (MIHS), along with better performance in timed up-and-go (TUG) and 4 m gait speed through a TKD intervention compared to an inactive CG. These results are similar to those reported by Lee, et al. [7] in older females with stage-2 hypertension, who showed a significant increase in MIHS and leg strength in a TKD intervention compared to an inactive CG. In the study by Valdés-Badilla, et al. [8], in apparently healthy older females, significantly higher performance was presented through a TKD intervention in the chair stand, arm curl, and 2-min step tests compared to multicomponent training (MCT). Both groups showed significant improvements in the general health dimension of HRQoL and postural balance. 

Taking into account that the deterioration of postural balance during aging leads to a higher fall risk, which is considered the second cause of loss of functionality independence in older people [9], obtaining improvements in postural balance in older people leads to essential benefits in functionality, not only by reducing the fall risk, but also by reducing disability and hospitalization, and increasing the degree of independence and HRQoL [9]. Among the most used physical activity strategies are MCT, resistance training, endurance, and walking exercise (WE), which have shown significant improvements in balance, measured through the Berg balance scale, the Activities-specific Balance Confidence scale, TUG, Falls Efficacy Scale—International [10,11], the HRQoL through Health Survey Short Form (SF-36), and the Brief Quality-of-life Survey the Euroquol-5D (EQ-5D), among other assessments [4]. However, these studies differ in their randomized and non-randomized controlled trial designs, which may differ in the evidence reported at the clinical level [12]. 

Additionally, the interventions using TKD in randomized controlled trials have had positive effects on physical performance and HRQoL variables compared to an inactive CG [5,6,7] and conventional physical activity strategies such as MCT in older people; it is a novel, practical, and easily accessible alternative [8]. These TKD interventions, when compared to other physical activity strategies, have only been two-arm randomized controlled trial designs. Three-arm or multi-arm randomized controlled trials provide greater transparency and accuracy in clinical reporting [13,14]. In this sense, the present study aimed to assess and compare the effects of a TKD program concerning MCT, WE, and an inactive CG on blood pressure, morphological variables (body mass index, BMI; body fat percentage, and fat-free mass), frequency of food consumption (healthy foods and unhealthy foods habits), cognitive status, HRQoL, physical fitness, MIHS, and postural balance in independent older females. Based on our preliminary study [8] and previous studies [5,6,7], we hypothesized that TKD produces significantly better HRQoL and postural balance effects than an MCT, WE, and CG. 

## 2. Methods

### 2.1. Study Design

There is a randomized controlled trial in this study, repeated measures, double-blinded participants and evaluators, and a quantitative approach for four parallel groups: TKD group, MCT group, WE group, and inactive CG. The randomization process was conducted using the research randomizer website (https://www.randomizer.org, accessed on 1 March 2023). CONSORT standards [15], TKD and Aging Project research procedure [16], and preliminary outcomes of the project [8] were used as the methodology. Additionally, the project has been registered in the United States’ ClinicalTrials.gov (code: NCT05275140; https://clinicaltrials.gov/search?cond=NCT05275140, obtained on 5 August 2024). The duration of the interventions was 16 weeks (48 sessions), including three weekly sessions on Mondays, Wednesdays, and Fridays lasting sixty minutes each. Blood pressure, morphological variables (BMI, body fat percentage, and fat-free mass), frequency of food consumption (healthy foods and unhealthy food habits), cognitive status, HRQoL, physical fitness, MIHS, and postural balance were all assessed. Every measurement was carried out at the same site (sports center and social headquarters) and in the afternoon, from 14:00 to 16:00, with the factors under control, including the temperature, and the evaluators who carried out the pre- and post-measures. Without musculoskeletal and/or cardiorespiratory injuries during the intervention, the older females did not exhibit pain before assessments or during training sessions.

### 2.2. Participants

**Figure 1:**
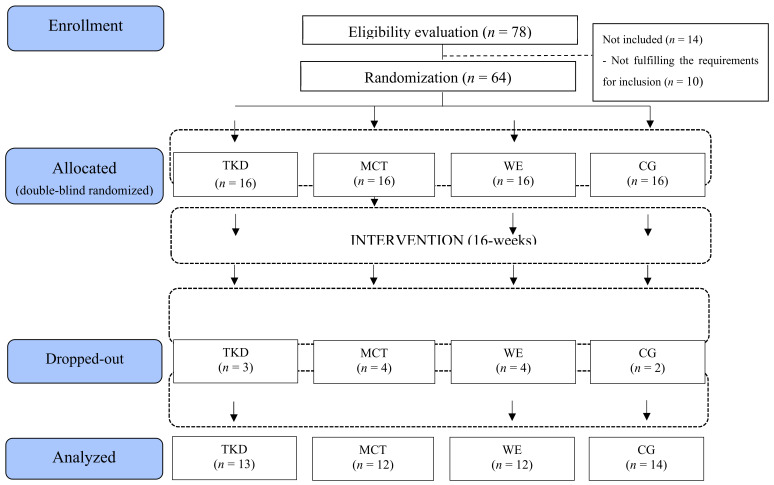
Study flowchart. Legends: CG: control group (no intervention). MCT: multicomponent training. TKD: adapted taekwondo. WE: walking exercise.

Sixty-four older females took part in the intervention at first. The optimal number of participants per group, according to the sample size calculation, was 16. The statistical power was calculated using the GPower software (Version 3.1.9.6, Franz Faul, Universiät Kiel, Kiel, Germany). According to a previous study [17], the minimum difference needed for significant clinical relevance was determined to be an average difference of 3.46 repetitions (chair stand test) with a standard deviation of 3.38 repetitions, taking into account an alpha level of 0.05 with 90% power and an anticipated loss of 15%. The following were the requirements for inclusion: (i) females between the ages of 60 and 65; (ii) those who demonstrated the capacity to comprehend and carry out instructions in a contextualized way through basic directives; (iii) those who were independent, as indicated by a score of at least 43 points on the Chilean Ministry of Health’s Preventive Medicine Exam for the Older People [18]; and (iv) those who were able to meet the intervention’s attendance requirement of at least 85%. The following were taken into consideration in relation to the exclusion criteria: (i) suffering from a disability; (ii) undergoing physical rehabilitation or having musculoskeletal injuries that prevent them from performing their regular physical activities; and (iii) being permanently or temporarily unable to participate in physical activity. Thirteen older females did not meet the minimum attendance or did not participate in the final assessments, specifically, 8 due to lack of time (n = 2 of TKD, n = 3 of MCT, and n = 3 of WE), 2 due to assuming care responsibilities (n = 1 of TKD and, n = 1 of MCT), and 3 due to not participating in the re-assessments (n = 1 of WE and n = 2 of CG). Thus, 13 older females in TKD (mean age: 63.08 ± 2.11 years; body mass: 62.26 ± 3.64 kg; bipedal height: 1.57 ± 0.05 m), 12 in MCT (mean age: 63.38 ± 1.78 years; body mass: 66.05 ± 2.77 kg; bipedal height: 1.57 ± 0.04 m), 12 in WE (mean aged: 64.21 ± 1.18 years; body mass: 61.83 ± 4.42 kg; bipedal height: 1.55 ± 0.04 m), and 14 in CG (mean age: 62.75 ± 1.66 years; body mass: 63.11 ± 4.75 kg; bipedal height: 1.59 ± 0.04 m) were analyzed in this study. Figure 1 outlines the condensed inclusion requirements.

By signing an informed permission form allowing the use of the data for scientific reasons, each participant acknowledged their acceptance of the inclusion criteria for handling and utilizing the data. The protocol was created in accordance with the Declaration of Helsinki and approved by the Scientific Ethics Committee of the Universidad Católica del Maule in Chile (number: N° 29-2022). 

### 2.3. Primary Outcomes

#### 2.3.1. Blood Pressure

In order to assess the systolic and diastolic blood pressure, an automated pressure monitor (08A, CONTEC, Germany) was acquired. The older females were evaluated after emptying their bladders and resting for at least ten minutes in a seated position with their backs, arms, and legs uncrossed. To determine which component had the greatest blood pressure, the initial evaluation was performed in both arms. The highest blood pressure in the arm was then subjected to two assessments (typically the dominant arm), with a third measure added if the difference between the results was greater than five mmHg. Blood pressure measurement procedures were performed in accordance with Reddy et al.’s [19] recommendations.

#### 2.3.2. Morphological Variables

Body weight was measured using a stadiometer (Seca 220, Germany; accuracy of 0.1 cm) and a digital scale (Seca 769, Germany; accuracy of 0.1 kg) to measure bipedal height. The BMI for each older female was also calculated by dividing bipedal height in m^2^ by body weight in kg. Every assessment was performed by a level II anthropometrist certified by the International Society for the Advancement of Kinanthropometry (ISAK) in accordance with its rules [20]. To determine the proportion of body fat percentage and fat-free mass, an eight-electrode tetrapolar bioimpedance device (InBody 570^®^, Body Composition Analyzers, Seoul, South Korea) was utilized. 

#### 2.3.3. Frequency of Food Consumption

For this, a modified version of an older adults’ eating habits survey was utilized, validated using 25 nutrition experts’ opinions, to inform the Delphi method [21]. There were two self-application sections in the survey’s design: (i) the initial (healthy food habits) section consists of 12 questions with a Likert scale score of 1 at the lowest and 5 at the highest, reflecting the prevalence of nutritious foods, such as the regularity of consuming advised food types, from avoiding them (1 point) to the recommended daily/weekly portions (5 points), with a rating based on the answers that range between 12 and 60 points (better eating habits, higher value) [21]; and (ii) the second section (unhealthy food habits) includes seven items, harmful foods or food groups (such as sugary drinks, alcohol, fried meals, fast food, sweet snacks, and coffee) that have been linked to the development of chronic non-communicable diseases. The scores for 6 of the questions are identical to those of the preceding one (1, does not consume, to 5, more than three servings daily or weekly), and one’s salt rating ranges from 1 to 3, reaching a value that ranges between 7 and 33 points; foods with higher scores are considered harmful. A poor eating habit is added, like seasoning food with salt without tasting it [21].

#### 2.3.4. Cognitive Status

The memory, phonetic fluency, and temporal-spatial orientation survey (MEFO in Spanish) [22] will be used to assess this in order to identify cognitive decline in older females. Participants in this poll are divided into three categories: those who have mild cognitive impairment, those who have cognitive impairment, and those who do not. The MEFO has the benefit of being simple and quick to use, and it is not impacted by educational attainment [22]. The MEFO evaluates temporal-spatial orientation, deferred-free recall, and phonetic fluency with the letter P. It is also validated for use with older people in Chile and has good sensitivity in the areas mentioned [22].

#### 2.3.5. Health-Related Quality of Life (HRQoL)

The SF-36, version 2, was used to collect this questionnaire. It assesses eight health aspects’ characteristics [23]: physical function, physical role, body pain, general health, vitality, social function, emotional role, and mental health. The questions from each dimension are combined to create a scale, where 100 represents the best health state and 0 represents the worst health status [23].

#### 2.3.6. Physical Fitness

The physical fitness was assessed using the Senior Fitness Test [24]. The 30 s chair stand test was used as the battery’s initial test, which counts the number of repetitions performed in 30 s to gauge lower body muscle strength. Using a 3-pound dumbbell and counting the number of repetitions completed in 30 s, the arm curl test assessed upper body muscle strength. A 2-min step test that counts the quantity of knee lifts that each participant performs to at least a 70-degree angle on their hip joint is used to measure cardiorespiratory fitness. The lower body degree of flexibility is measured in cm during the chair sit-and-reach test. The back scratch test assesses upper body flexibility in centimeters. The TUG measures agility and dynamic balance by encircling a cone at 8 feet (2.44 m) while managing time in seconds.

#### 2.3.7. Maximal Isometric Handgrip Strength (MIHS)

Using a hydraulic dynamometer (Camry, model EH101, Zhongshan, China), the MIHS was measured following previous suggestions [25]. The older females were sitting with their elbows flexed and shoulders abducted 90 degrees to one side of their body, with their wrists neutral and their forearms in a neutral alignment. The hand’s dimensions considered when configuring the dynamometer; it should be possible to hold the device comfortably and functionally while maintaining sufficient closure of the metacarpal and interphalangeal joints in the first position, which favors contact between the thumb and index first phalanges. Every individual participated in three tries on each hand, followed by a pause period of 120 s, before analyzing the best of the three attempts.

#### 2.3.8. Postural Balance

Using a force platform (ArtOficio Ltd., Valparaíso, Chile), the center of pressure’s displacement was measured, following earlier recommendations [26]. The sampling rate used to collect the data was 40 Hz. Both eyes closed (EC) and eyes open (EO) were used to measure postural balance, and each assessment lasted 30 s. The older females were told to keep their feet roughly shoulder-width apart, their arms at their sides, and their bodies as still as possible while walking on two feet. Matlab r2012a (Mathworks Inc., Natick, MA, USA) was used to compute the center of pressure’s area and velocity variables.

### 2.4. Secondary Outcomes

Additionally, baseline measures of civil status (married, separated, widowed, single, etc.), academic level (primary, secondary, bachelor’s or postgraduate), age (years), and smoking status (yes or no) were gathered.

### 2.5. Intervention

**Figure 2:**
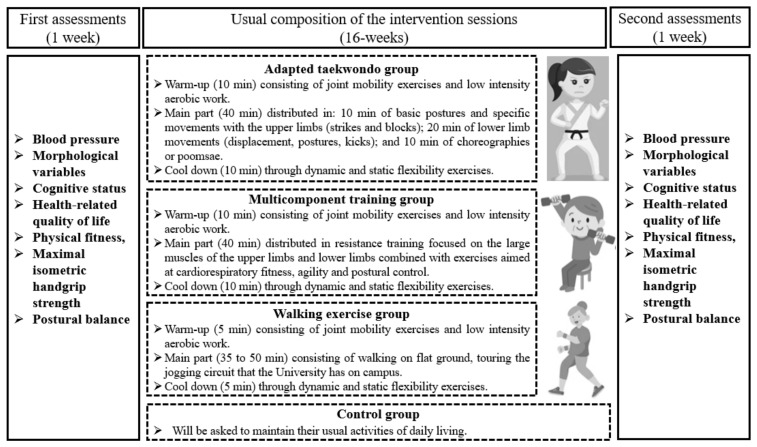
Measurements and regular sessions of the intervention.

All programs (TKD, MCT, and WE) lasted 16 weeks (48 sessions), and the interventions were developed according to the study protocol [16]. The TKD and MCT’s overall structure programs included a 10-min warm-up that involved low-intensity cardiovascular activity and joint mobility exercises; after that, for 40 min, the main part (TKD or MCT) was created to complete with a 10-min cooldown through exercises for both static and dynamic flexibility, which is explained in detail in a preliminary study [8]. For their part, the WE protocol was disseminated every other day in three weekly sessions lasting 45 to 60 min. Version 1.3 of the Polar Team app (Polar Electro Oy, Kempele, Finland) consistently monitored the older females. The intensity of the interventions (TKD, MCT, and WE) utilized a heart rate sensor strap (H10, Polar Electro Oy, Kempele, Finland) that was live transmitted via Bluetooth to a tablet (iPad 4, Apple, Inc., Cupertino, CA, USA) to measure each older female’s maximum heart rate (HRmax), which was kept between 50% and 70% of HRmax as a control. A National Sports Federation of Taekwondo WT-certified taekwondo instructor (for TKD) and Master’s students in physical activity sciences with expertise in training older people (MCT and WE) led the sessions. Figure 2 provides a summary of the intervention’s evaluations and scheduled sessions.

The TKD program was non-contact exercises (main part), which were broken up into 10 min of fundamental positions and particular technical foundations with the upper body (blocks and strikes) and 20 min of lower body technical foundations (movement, positions, and kicks), performed individually and in pairs with and without the use of taekwondo impact pads and shields; also, specific choreographies or *poomsae* (*Kibom Poomsae and Il Jang*) were performed for 10 min [8]. During the first four weeks of training, there were three sets of eight repetitions of upper and lower limbs specific technical foundations, with a two-minute rest between sets [8,16]. Weeks 5 through 8 saw an increase in volume, to 4 sets of 8 repetitions per specific technical foundations, with a two-minute break in between sets. The sets stayed at four throughout weeks 9 and 12, while the repetition count was raised to 12. Lastly, the rest period was cut to 90 s between weeks 13 and 16 while the number of sets and repetitions (4 × 12) is maintained. During the 16 weeks the *poomsae* were performed in 6 repetitions per session [16].

Elastic bands, poles, 2 kg medicine balls, and chairs were used in the 40-min circuit that comprised the MCT program (main part), which included exercises for agility, postural balance, and cardiorespiratory fitness. The exercises target the quadriceps, hamstrings, glutes, and gastrocnemius muscles as well as the big muscle groups of the upper body (biceps, triceps, deltoids, and latissimus dorsi). Three sets of 10 repetitions of each muscle activity, separated by a two-minute rest period, made up the first training volume (the first four weeks). Slow motions were employed, with concentric contraction lasting 2 s and eccentric contraction lasting 4 s. The volume increased to 4 sets of 10 repetitions each muscle exercise with 2 min of rest in between sets during weeks 5 and 8. The sets stayed at 4 throughout weeks 9 and 12, while the repetition count was raised to 12. Lastly, the rest period was cut to 90 s between weeks 13 and 16 while the number of sets and repetitions (4 × 12) was maintained. The OMNI-Resistance Exercise Scale of Perceived Exertion was employed to regulate the level of the resistance training, which varied from mild to intense (5 to 8 points) [27]. The intensity of cardiorespiratory fitness, agility, and postural control through perceived exertion was measured using Borg’s 10-point scale [28], and the heart rate sensor (H10, Polar Electro Oy, Kempele, Finland) was used to manage it.

A 5-min warm-up consisting of joint mobility and flexibility exercises was included in the WE program. The main part was then developed for 35 to 50 min (with an increase of 5 min every 4 weeks). This included walking on level ground and a 5-min cool down that included both static and dynamic flexibility exercises. The intensity was kept moderate to vigorous (HRmax ranging from 50% to 70%) and managed via a strap with a heart rate sensor (H10, Polar Electro Oy, Kempele, Finland). Every 4 weeks, the load progression was modified, resulting in a 5 min walk increase and individual cadence regulation, increasing the distance the participants covered. The intensity controlled through perceived exertion was measured using Borg’s 10-point scale [28] and controlled with a heart rate sensor strap (H10, Polar Electro Oy, Kempele, Finland).

In reference to the CG, the older females participated in the initial and final measures and were instructed to continue with their regular daily routines. Once a week, a research assistant called the participants to check on their activities and ask about their health. 

### 2.6. Statistical Analysis

The statistical software GraphPad Prism 9 (GraphPad Software, Inc., La Jolla, CA, USA) was used to do the analysis. The data are displayed using the mean and standard deviations. The results of the analysis met the Shapiro–Wilk test for data normality. The time × group effect of each variable was then assessed using a two-factor mixed analysis of variance (ANOVA) model with repeated measures. When the time × group interaction was significant, the intragroup (pre vs. post) and intergroup (TKD vs. MCT vs. WE vs. CG) differences were assessed using a Bonferroni multiple comparison test (post hoc). The effect size of the time × group interaction was computed using the partial eta square (ηp^2^), and the results were interpreted with respect to the ηp^2^ values of 0.01, 0.06, and 0.14 [29], which represent small, moderate, and large effect sizes (ESs), respectively. Cohen’s d was used to determine the ES for multiple comparisons, taking into account a small (≥0.2), moderate (≥0.5), or large (≥0.8) effect [30]. At 5%, a significant difference was determined for each analysis.

## 3. Results

### 3.1. Blood Pressure, Morphological Variables, and Frequency of Food Consumption

The blood pressure, morphological variables, and frequency of food consumption assessments did not show significant intragroup and intergroup interactions. These assessments were systolic blood pressure (F_(3,55)_ = 1.84; *p* = 0.171), diastolic blood pressure (F_(3,55)_ = 1.73; *p* = 0.192), BMI (F_(3,55)_ = 2.62; *p* = 0.078), body fat percentage (F_(3,55)_ = 0.76; *p* = 0.525), fat-free mass (F_(3,55)_ = 1.84; *p* = 0.172), healthy foods habits (F_(3,55)_ = 0.56; *p* = 0.646), and unhealthy foods habits (F_(3,55)_ = 1.45; *p* = 0.257).

### 3.2. Cognitive Status

**Figure 3:**
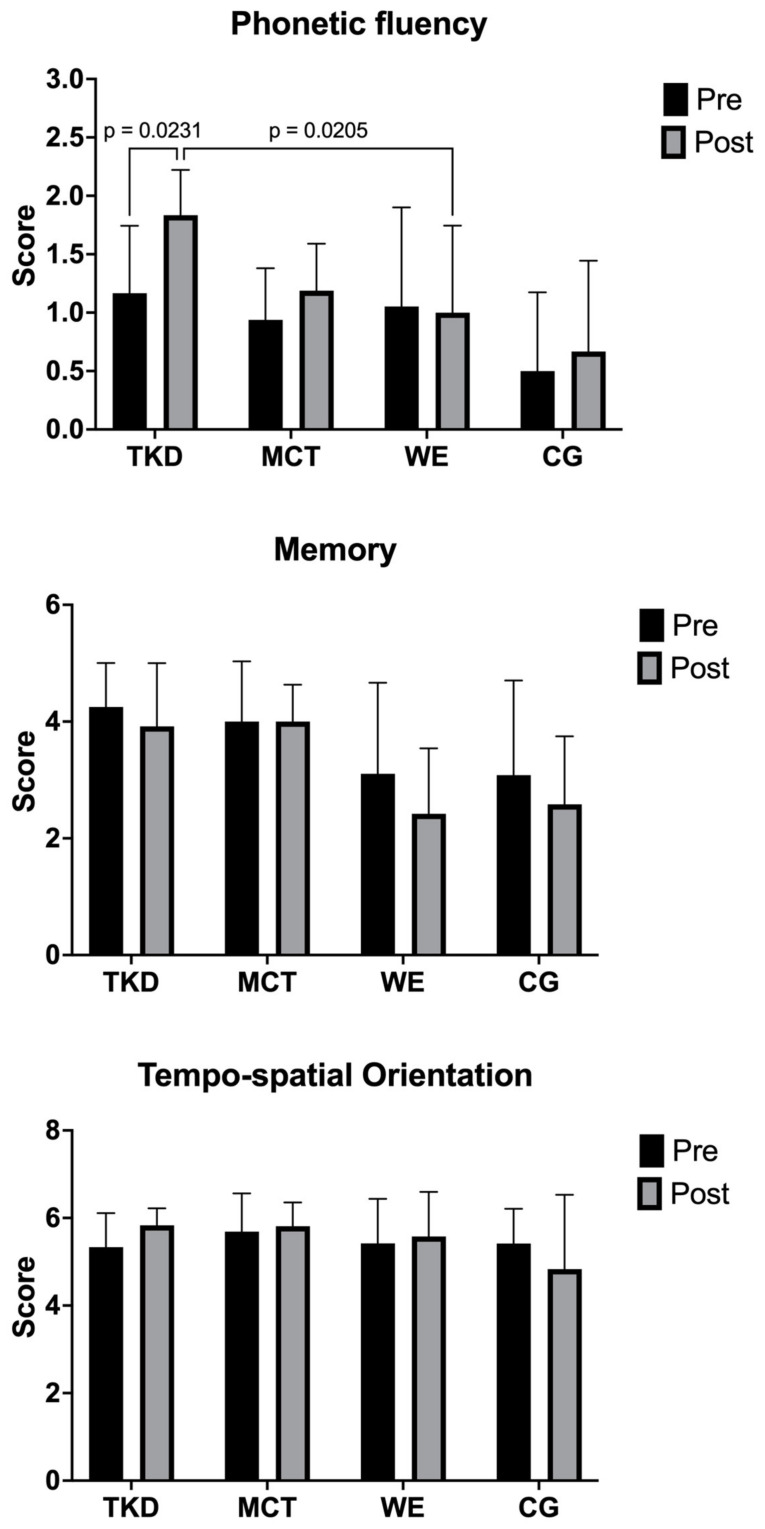
Multiple comparisons of cognitive status assessment of adapted taekwondo, multicomponent training, walking exercise, and control groups. Legends: TKD: adapted taekwondo. MCT: multicomponent training. WE: walking exercise. CG: control group.

The two-way mixed ANOVA test revealed a significant time × group interaction only for phonetic fluency (F_(3,55)_ = 3.17; *p* = 0.046; ηp^2^ = 0.147, *large effect*), where an increase in the TKD group was observed after the intervention (*p* = 0.023; ES = 1.35, *large effect*) and significant differences when comparing the TKD vs. WE groups (*p* = 0.021; ES = 1.89, *large effect*) (Figure 3). For the dimensions of memory (F_(3,55)_ = 0.98; *p* = 0.419; ηp^2^ = 0.047, *small effect*) and tempo-spatial orientation (F_(3,55)_ = 1.28; *p* = 0.288; ηp^2^ = 0.052, *small effect*), no significant time × group interaction was observed.

### 3.3. Health-Related Quality of Life (HRQoL)

**Figure 4:**
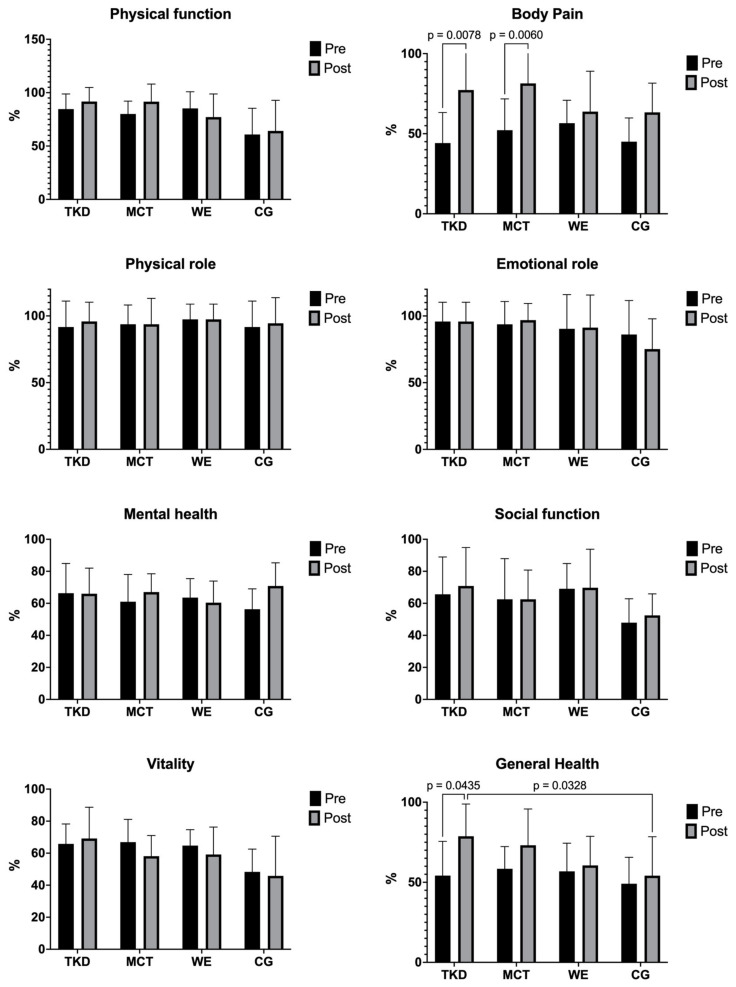
Multiple comparisons of health-related quality of life dimensions of adapted taekwondo, multicomponent training, walking exercise, and control groups. Legends: TKD: adapted taekwondo. MCT: multicomponent training. WE: walking exercise. CG: control group.

Table 1 shows the HRQoL dimensions before and after intervention results for TKD, MCT, WE, and CG. The two-way mixed ANOVA test revealed a significant time × group interaction for body pain (F_(3,55)_ = 3.31; *p* = 0.041; ηp^2^ = 0.122, *large effect*) and general health (F_(3,55)_ = 2.27; *p* = 0.007; ηp^2^ = 0.084, *large effect*). Multiple comparisons of these results are presented in Figure 4. In body pain, significant increases in HRQoL were found in both the TKD (*p* = 0.008; ES = 1.46, *large effect*) and MCT (*p* = 0.006; ES = 0.80, *large effect*) groups, while intergroup comparisons revealed no differences between interventions. On the other hand, in general health, an increase in HRQoL was evidenced in the TKD group (*p* = 0.044; ES = 1.18, *large effect*). In the intergroup comparison, a difference was observed between TKD and CG (*p* = 0.033; ES = 1.11, *large effect*).

### 3.4. Physical Fitness and Maximal Isometric Handgrip Strength (MIHS)

Table 2 shows the physical fitness and MIHS assessments’ before and after intervention results for TKD, MCT, WE, and CG. The two-way mixed ANOVA test revealed a significant time × group interaction for the 30 s chair stand (F_(3,55)_ = 4.25; *p* = 0.017; ηp^2^ = 0.179, *large effect*), arm curl (F_(3,55)_ = 8.51; *p* < 0.001; ηp^2^ = 0.299, *large effect*), chair sit-and-reach (F_(3,55)_ = 8.56; *p* < 0.001; ηp^2^ = 0.297, *large effect*), TUG (F_(3,55)_ = 5.88; *p* = 0.004; ηp^2^ = 0.220, *large effect*), and MIHS non-dominant hand (F_(3,55)_ = 4.53; *p* = 0.013; ηp^2^ = 0.194, *large effect*). On the other hand, for the 2-min step test (F_(3,55)_ = 0.42; *p* = 0.737; ηp^2^ = 0.022, *medium effect*), back scratch (F_(3,55)_ = 0.59; *p* = 0.625; ηp^2^ = 0.028, *medium effect*), and MIHS dominant hand (F_(3,55)_ = 0.85; *p* = 0.480; ηp^2^ = 0.044, *medium effect*), there was no significant interaction.

**Figure 5:**
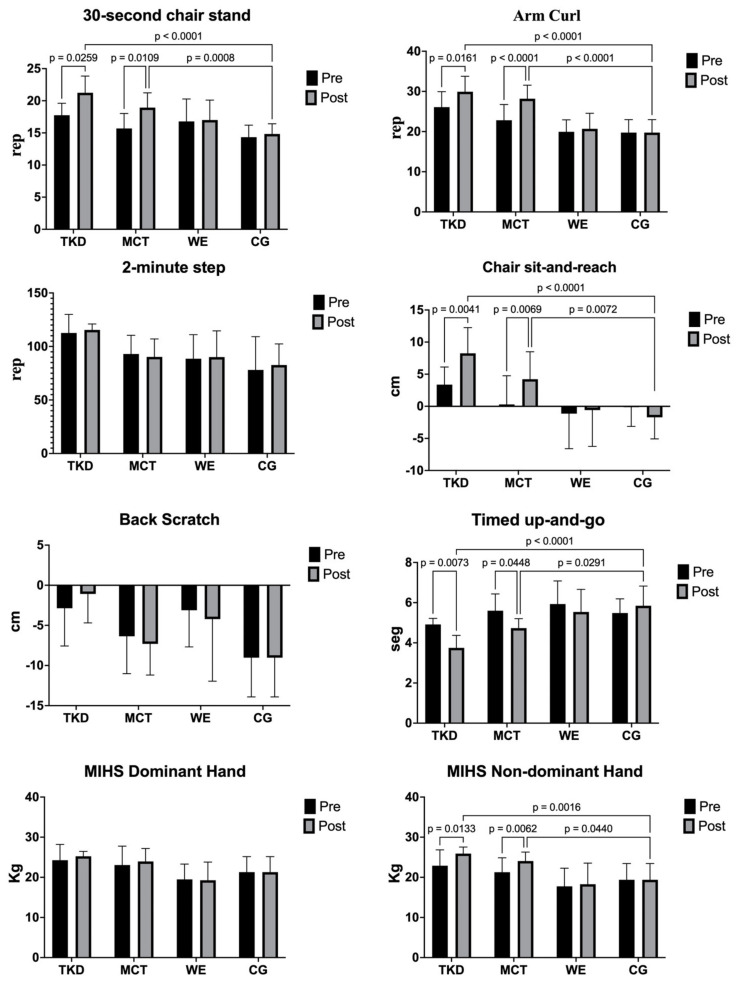
Multiple comparisons of physical fitness assessments of adapted taekwondo, multicomponent training, walking exercise, and control groups. Legends: TKD: adapted taekwondo. MCT: multicomponent training. WE: walking exercise. CG: control group. MIHS: maximal isometric handgrip strength.

The results of the multiple intragroup and intergroup comparisons are shown in Figure 5. Regarding the 30 s chair stand test, significant increases in the number of repetitions were found in TKD (*p* = 0.026; ES = 1.53, *large effect*) and MCT (*p* = 0.011; ES = 1.39, *large effect*) after the intervention. Similar results were observed in the arm curl test, where the number of repetitions increased for the TKD (*p* = 0.016; ES = 1.01, *large effect*) and MCT (*p* < 0.001; ES = 1.47, *large effect*) groups after the intervention. In the chair sit-and-reach test, significant gains in lower limb flexibility were observed for the TKD (*p* = 0.007; ES = 1.43, *large effect*) and MCT (*p* = 0.045; ES = 0.88, *large effect*) groups. Likewise, in the MIHS non-dominant hand, a significant increase in muscle strength was observed for both the TKD (*p* = 0.013; ES = 0.98, *large effect*) and the MCT (*p* = 0.006; ES = 0.94, *large effect*) groups. 

The intergroup comparison revealed significant differences between TKD and CG in the 30 s chair stand (*p* < 0.001; ES = 3.01, *large effect*), arm curl (*p* < 0.001; ES = 2.87, *large effect*), chair sit-and-reach (*p* < 0.001; ES = 2.69, *large effect*), TUG (*p* < 0.001; ES = 2.42, *large effect*), and MIHS non-dominant hand (*p* = 0.002; ES = 2.08, *large effect*) tests. Similarly, significant differences between MCT and CG were observed in the 30 s chair stand (*p* < 0.001; ES = 2.54, *large effect*), arm curl (*p* < 0.001; ES = 3.01, *large effect*), chair sit-and-reach (*p* = 0.007; ES = 1.52, *large effect*), TUG (*p* = 0.029; ES = 1.39, *large effect*), and MIHS non-dominant hand (*p* = 0.044 ES = 1.42, *large effect*) tests.

### 3.5. Postural Balance

Table 3 shows the center of pressure variables’ before and after intervention results for TKD, MCT, WE, and CG. The two-way mixed ANOVA test revealed a significant time × group interaction for area EO (F_(3,55)_ = 5.32; *p* = 0.007; ηp^2^ = 0.222, *large effect*), mediolateral velocity EO (F_(3,55)_ = 4.34; *p* = 0.016; ηp^2^ = 0.191, *large effect*), area EC ((F_(3,55)_ = 4.07; *p* = 0.021; ηp^2^ = 0.167, *large effect*), anteroposterior velocity EC (F_(3,55)_ = 6.50; *p* = 0.003; ηp^2^ = 0.241, *large effect*), and mediolateral velocity EC (F_(3,55)_ = 6.43; *p* = 0.003; ηp^2^ = 0.213, *large effect*).

**Figure 6:**
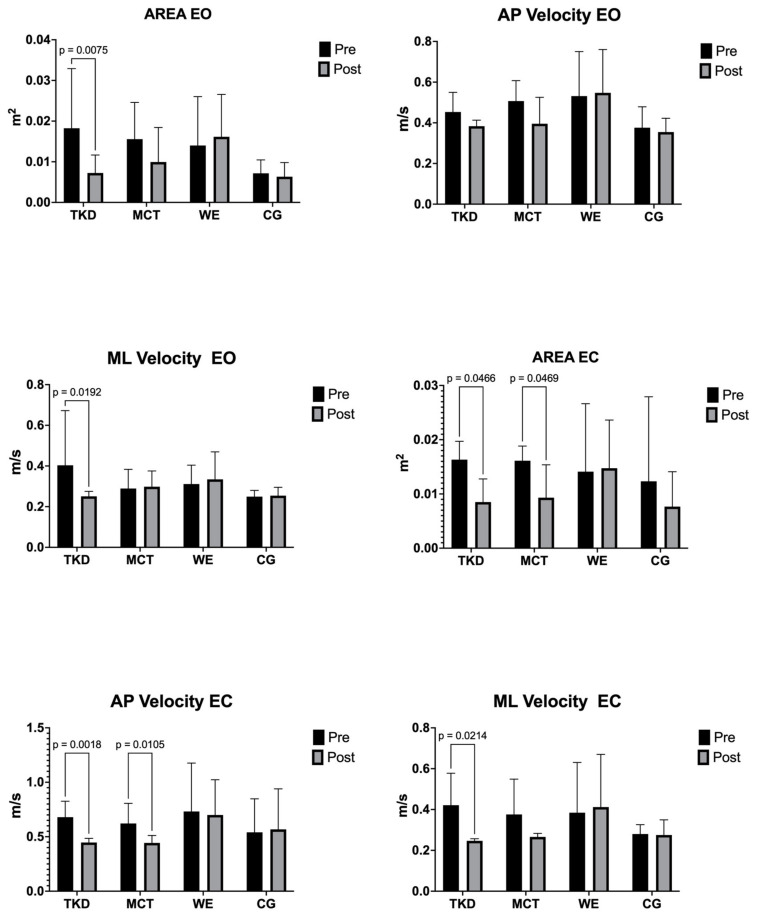
Multiple comparisons of postural balance variables (center of pressure) of adapted taekwondo, multicomponent training, walking exercise, and control groups. Legends: TKD: adapted taekwondo. MCT: multicomponent training. WE: walking exercise. CG: control group. EO: eyes closed. EC: eyes closed. AP: anteroposterior. ML: mediolateral.

The results of the multiple intragroup and intergroup comparisons are shown in Figure 6. After the intervention in EO, an improved postural balance was observed only in the TKD group in the area (*p* = 0.008; ES = 1.00, *large effect*) and the mediolateral velocity (*p* = 0.019; ES = 0.79, *medium effect*). In the EC area, postural balance was improved in the TKD (*p* = 0.047; ES = 1.97, *large effect*) and MCT (*p* = 0.047; ES = 1.47, *large effect*) groups. Likewise, in anteroposterior velocity EC, both the TKD (*p* = 0.002; ES = 2.16, *large effect*) and MCT (*p* = 0.011; ES = 1.28, *large effect*) groups experienced an improvement in postural balance. Finally, only a significant improvement was found in the TKD group for mediolateral velocity EC (*p* = 0.021; ES = 1.57, *large effect*). No significant differences were detected between the training groups.

## 4. Discussion

This study aimed to assess and compare the effects of a TKD program concerning MCT, WE, and CG on blood pressure, morphological variables, frequency of food consumption, cognitive status, HRQoL, physical fitness, MIHS, and postural balance in independent older females. The main results were as follows: (i) TKD group obtained a significantly higher value post-intervention in phonetic fluency than WE; (ii) the TKD group showed greater improvement in the body pain dimension of HRQoL than the CG, while both TKD and MCT groups achieved significantly higher intragroup results post-intervention in the general health dimension of HRQoL, with no significant differences between groups; (iii) TKD and MCT groups achieved significantly greater results in the 30 s chair stand, arm curl, chair sit-and-reach, TUG, and MIHS non-dominant hand tests regarding the CG; (iv) Only the TKD group significantly improved postural balance in EO for the area and mediolateral velocity, and the mediolateral velocity EC, while the TKD and MCT groups significantly improved in area and anteroposterior velocity EC. Therefore, our hypothesis is corroborated.

### 4.1. Blood Pressure, Morphological Variables, and Frequency of Food Consumption

No significant intragroup or intergroup changes were reported in systolic and diastolic blood pressure and morphological variables such as BMI, body fat percentage, and fat-free mass. These results are similar to those reported by Valdés-Badilla, et al. [8], in apparently healthy older females, where there were no significant improvements in time × group interactions in BMI, body fat percentage, fat-free mass, healthy food, unhealthy food, systolic and diastolic blood pressure through TKD and MCT interventions with a duration of 8 weeks with 3 sessions a week of 60 min at intensities between 50–70% HRmax. However, in the study by Lee, et al. [7] in older people with stage 2 hypertension, significant improvements in BMI (*p* = 0.037), systolic (*p* = 0.031), and diastolic (*p* = 0.013) blood pressure were reported by a TKD intervention for 12 weeks with a frequency of three sessions per week with a duration of 60 min at an intensity between 40 and 60% HRmax compared to an inactive CG. It is important to emphasize that in the study by Lee, et al. [7], the sample was composed of postmenopausal older females with stage 2 hypertension, who may have been more susceptible to the TKD intervention to improve BMI and systolic and diastolic blood pressure. Regarding our findings, these can be attributed to the eating habits, since the participants did not follow a dietary guideline that complemented their training. Evidence indicates that physical activity plus implementing a nutritional plan that provides an optimal protein intake (1.0 to 1.2 g/kg body weight/day) can improve body composition in older people [31,32]. Since eating habits are modifiable, generating nutritional education through a multidisciplinary team of nutrition professionals is essential. Reducing ultra-processed foods may help lower older people’s systolic and diastolic blood pressure [33]. In this regard, it has been reported that minimal reductions in systolic blood pressure (1 mm Hg) can significantly prevent cardiovascular death [34]. Therefore, to improve body composition, frequency of food consumption, and systolic and diastolic blood pressure in older people, it is advisable to perform TKD interventions incorporating a nutritional plan for participants.

### 4.2. Cognitive Status

In the cognitive status, there were significant improvements in phonemic fluency in favor of TKD regarding WE. Similar results were reported by Welford, et al. [35] in a three-arm RCT of yoga and aerobic exercise interventions lasting 12 weeks with a frequency of three sessions per week of 60 min duration showing significant improvements in verbal fluency in yoga (*p* = 0.002) and aerobic exercise (*p* = 0.004) compared to an inactive CG in physically inactive older people [35]. However, in the present study, no significant improvements in memory and tempo-spatial orientation were reported in the groups analyzed. Different results to those reported by Jansen, et al. [36] in apparently healthy older people showing significant improvements in memory (*p* < 0.001) through a karate intervention compared to mindfulness training lasting 8 weeks with a total of 15 sessions per week of 75 min duration. The literature has defined neurotrophic growth factors as one mechanism that leads to improved brain function through regular physical activity [5]. It has been proposed that when skeletal muscle contracts, it secretes various proteins into the circulation known as myokines [5]. Subsequently, these myokines could elevate neurotrophic factors such as irisin, brain-derived neurotrophic factor (BDNF), and insulin-like growth factor (IGF-1) [37]. These factors are important in hippocampal plasticity and long-term memory [38,39]. Although we did not find improvements in memory and tempo-spatial orientation in the groups analyzed, research has reported increased serum levels of BDNF and IGF-1 through interventions with TKD [5,40], although this was not measured in our study. Improvements in phonemic fluency in favor of TKD compared to WE can be attributed to neurobiological and psychological processes [35]. First, it has been reported that exercise can regulate the hypothalamic–pituitary–adrenal axis, leading to reductions in stress hormones [41]. As a secondary effect, reducing circulating stress hormones can ameliorate the negative effects of chronic stress on cognitive function. TKD in older people can improve self-esteem, confidence, and motivation compared to WE [5,42]. These psychological effects may distract from stressful life events and reduce negative attentional biases in older people [5,42].

### 4.3. Health-Related Quality of Life (HRQoL)

Regarding HRQoL, significant improvements were found in favor of TKD for the body pain and general health dimensions compared to the other interventions. These were similar results to those reported by Valdés-Badilla, et al. [8] in older females showing significant improvements through TKD in general health (*p* < 0.001) as well as MCT (*p* = 0.013). However, both groups had no significant improvements in body pain (*p* = 0.161). In contrast, in the present study, in the dimensions of physical function, physical role, vitality, social function, emotional role, and mental health, there were no improvements for the groups analyzed. These are similar results to those presented by Valdés-Badilla, et al. [8] in older females, where no significant improvements were found through interventions with TKD and MCT in the dimensions of physical function (*p* = 0.78), physical role (*p* = 0.99), vitality (*p* = 0.38), social function (*p* = 0.84), and emotional role (*p* = 0.99). The implications of HRQoL for older people are manifested at both individual and societal levels [43]. Better HRQoL translates into greater independence, well-being, and self-sufficiency in activities of daily living [3]. Socially, it may reflect the community’s care and respect for older people, which may have economic repercussions by decreasing health care costs for families and public resources. Our findings are consistent with existing evidence that has reported positive changes with a small and moderate ES for HRQoL in middle-aged and older people [4]. Following a TKD intervention in older people, a decrease in depression levels has also been reported, which may translate into better general health of the participants [6]; this is relevant, given that general health has been related to some morphological variables and physical fitness factors that increase the probability of having a low HRQoL [44]. For example, body mass, waist circumference, back scratch test, and TUG were significantly associated with low mental and general health dimensions of HRQoL in physically active older females [44]. The concept of HRQoL in older people contains different dimensions, such as physical and mental health, levels of independence, social participation, personal beliefs, and environmental context [45]. The present study only reported improvements in the body pain and general health dimensions; this fact could reflect that additional follow-up may be necessary after the interventions to analyze changes in dimensions that may require additional time to improve.

### 4.4. Maximal Isometric Handgrip Strength (MIHS)

In MIHS, significant improvements were only reported for the non-dominant hand in TKD and MCT compared to the other groups. These results are similar to those reported by Kim, et al. [46] in older females with arterial hypertension, showing a significant increase in MIHS dominant hand (*p* = 0.01) through a TKD intervention lasting 12 weeks with a frequency of three sessions per week of 90 min duration compared to an inactive CG. Similarly, Lee, et al. [7] showed significant increases in MIHS dominant (*p* = 0.03) and non-dominant (*p* = 0.03) hands in older females with stage 2 arterial hypertension using TKD compared to an inactive CG. Similarly, Rodrigues, et al. [47], in apparently healthy older people, showed significant increases in MIHS for both hands (*p* = 0.03) through an MCT intervention for 20 weeks with a frequency of two sessions per week of 60 min compared to concurrent training. MIHS reflects various physical function parameters and is considered an important indicator of HRQoL in older people [48]. In this regard, it has been reported that decreased MIHS affects cognitive decline and dementia in older people [49]. The increase in MIHS in the TKD and MCT groups can be explained by the actions performed during the interventions. In TKD, the constant execution of techniques such as specific technical foundations with the upper body [8,16], in which participants spent a prolonged time exerting isometric force by clenching their fists, may have generated an increase in the forearm muscles, leading to improvements in the non-dominant hand, with a better stimulus probably being necessary to improve the dominant hand. In MCT, improvements may be explained by the execution of upper body exercises that focused on the biceps, triceps, and deltoids involving flexion and extension muscle actions at an intensity on the perceived exertion (Borg’s 10-point scale) of moderate to vigorous (5 to 8 points), which may have contributed to the increase in forearm strength and consequently MIHS [8].

### 4.5. Arm Curl Test

Significant improvements were reported in the present study in the TKD and MCT groups in the arm curl test. Similarly, de Queiroz, et al. [50] showed significant increases in arm curl (*p* < 0.05) in apparently healthy older males through a 12-week jiu-jitsu training intervention with a frequency of two sessions per week of 90 min compared to an inactive CG. Similar to that reported by Rodrigues, et al. [47], older people showed significant increases in arm curl (*p* < 0.001) using MCT compared to concurrent training. However, in the study by Valdés-Badilla, et al. [8], only significant increases (*p* < 0.0001) in arm curl were reported for TKD compared to MCT in older females. The arm curl test consists of performing the maximum number of repetitions of arm curls in 30 s, while the subjects are seated in a chair, assessing upper body strength-endurance. Strength-endurance is characterized by an individual’s ability to execute a number of repetitions of an exercise or a technical gesture for a given time or until muscular failure, maintaining the prescribed rhythm and the same efficiency [51]. In this sense, similarly to MIHS, the improvements in the TKD and MCT groups can be attributed to the repetitive efforts performed by TKD techniques (e.g., punching techniques) and to the upper body exercises performed with elastic bands and poles in MCT (e.g., four sets of ten repetitions of biceps curls). The longer the time of tension with a load that promotes increased muscle fatigue, the better the gains in strength endurance due to the great metabolic mobilization [51]. Therefore, technical actions with punches and upper body exercises targeting the biceps and triceps muscles may have increased forearm fatigue tolerance in older people, generating increases in the arm curl test.

### 4.6. 30 s Chair Stand Test

In the present 30 s chair stand test study, improvements were only presented in the TKD and MCT groups. These are similar results to those reported by Kim, et al. [46] in older females with arterial hypertension presenting significant increases (*p* < 0.01) in a 30 s chair stand using TKD compared to an inactive CG. Similarly, Rodrigues, et al. [47], in older people, showed significant increases in 30 s chair stand (*p* < 0.001) using MCT compared to concurrent training. However, Valdés-Badilla, et al. [8] only presented significant increases (*p* = 0.001) in the TKD group for the 30 s chair stand test compared to MCT in older females. Our findings may reflect the effectiveness of TKD and MCT in improving lower body muscle strength. In this sense, the improvements in the TKD group may be attributed to the variety of techniques using the lower body, which are characterized by applying significant force against the ground to transmit forces to the target [52]. In addition, techniques such as kicking constantly require hip flexion and knee joint flexion-extension [52], translating into older people making constant and repetitive efforts using their body weight. On the other hand, in the MCT group, exercises for the lower body were included, specifically for the quadriceps, hamstrings, glutes, and gastrocnemius muscle groups. The actions performed by the older people constantly involved flexion and extension movements of the lower body, which, due to its similarity to the 30 s chair stand test, could have generated increases in the test through an improvement in the neuromuscular function of the lower body, which has a key role in the eccentric and concentric phase of movements similar to the squat [47].

### 4.7. Flexibility

There were significant improvements for the TKD and MCT groups in lower body flexibility by chair sit-and-reach. These are similar results to those reported by Jofré-Saldía, et al. [53] in apparently healthy older people showing significant improvements in chair sit-and-reach (*p* = 0.04) in favor of an MCT intervention for 27 weeks with a frequency of three sessions per week of 45 min duration per session compared to an inactive CG. However, in the study by Valdés-Badilla, et al. [8], only significant improvements were reported in favor of a TKD intervention compared to MCT in chair sit-and-reach (*p* = 0.001) in older females. In this regard, the improvements in the chair sit-and-reach test in the TKD group can be attributed to the repetitive action of techniques such as kicking in conjunction with static stretching performed during the training sessions. Techniques that include kicking are similar to dynamic training to increase the range of motion [54]. Dynamic techniques are characterized by active movement within the range of motion of the joint involved in the sport technique [54]. For example, when older people performed some technique that included kicking, they performed a dynamic technique to increase flexibility. Wasik and Shan [55] have reported that during leg elevation in preparation for the kick, the slight flexion of the trunk in the direction of the kicking leg is responsible for increasing the range of motion of the hip joint, which would generate a pre-stretch of the hip extensor muscles and contribute to a greater height of the final kick. Conversely, the MCT group, through the execution of multi-joint exercises for the lower body, may have generated adaptations in the neuromuscular performance of older people, specifically a decrease in antagonistic coactivation and an increase in the reciprocal inhibition reflex, which would translate to a greater amplitude in the range of motion [56].

In flexibility for the upper body using the back scratch, no significant improvements were reported in favor of any group analyzed in the present study. On the contrary, Jofré-Saldía, et al. [53], in apparently healthy older people, reported significant improvements in back scratch (*p* = 0.000) in favor of MCT compared to an inactive CG. However, Valdés-Badilla, et al. [8], in older females, did not report significant improvements (*p* = 0.05) in back scratch in interventions using TKD and MCT. Based on our findings, increased training volume may be needed to improve upper body flexibility, complementing interventions with specific exercises that challenge the external and internal rotators of the shoulder joint over a wide range of motion, given that evidence indicates that it is possible to improve upper body range of motion in older people [53], possibly due to increased stretch tolerance, increased fascicle length, and changes in angle of pennation.

### 4.8. Postural Balance

Significant improvements were reported in the area and in the anteroposterior velocity EC for the TKD and MCT groups in the postural balance for the center of pressure. While in the area and mediolateral velocity of EO and mediolateral velocity of EC, significant improvements were only reported in TKD and anteroposterior velocity EO, and no significant improvements were reported in any group in the present study. In a pilot study conducted by Maria da Silva, et al. [57] in older people with low schooling, significant improvements in anteroposterior velocity EO (*p* = 0.004) were presented by an MCT intervention. However, in a study by Vieira, et al. [58] in apparently healthy older people, no significant improvements in anteroposterior and mediolateral velocity EO and EC were reported after a 12-week resistance training intervention compared to an inactive CG. For its part, in the study of Valdés-Badilla, et al. [8], no significant differences between the TKD and MCT interventions could be found; however, MCT only improved ML velocity EC (*p* = 0.0393), while TKD improved area EC (*p* = 0.0218), mean velocity EC (*p* = 0.0035), and ML velocity EC (*p* = 0.0002) conditions. TKD consists of various movements that combine the upper and lower body, including punching and kicking. These techniques require stabilizing the body to execute efficient movement and balance during dynamic actions. In addition, these movements involve long and wide stances that require older people to spend more time supporting a single limb as they lengthen their stride to achieve the correct stance. For example, it has been reported that through practicing TKD, the swing path and swing area of the lower body decreased significantly [59]. This fact may be related to the adjustment of the center of pressure, with higher plantar pressure that can provide better sensory stimulation to the mechanoreceptors [59], leading to an overall increase in neural feedback from the cutaneous receptors to the central nervous system, improving postural balance. MCT has been reported as a valuable strategy to improve fall rates and balance in older people [10]. However, TKD might be more favorable in improving postural balance in older people given the demands of movements that continuously require optimal motor control [59], incorporating afferent components and their efferent responses to maintain joint stability during sports techniques.

### 4.9. Timed Up-And-Go (TUG)

Only significant improvements in TKD and MCT were presented in the present study for the TUG test. Similar results to those presented by López-López, et al. [60] in institutionalized older people with a 12-week MCT intervention showed significant improvements in TUG (*p* < 0.001) compared to an inactive CG. Similarly, Baek, et al. [6], in older people with dementia and depression, reported significant improvements in TUG (*p* < 0.05) in favor of a 12-week TKD intervention compared to an inactive CG. Our findings may be attributed to the fact that TKD and MCT may have been more effective in improving the capacity of the lower body extensor muscles by increasing torque during gait. It has been reported that during gait with advancing age, step lengths become shorter, walking pace decreases, and the time in which both feet touch the ground increases, generating double support [61]. Therefore, TKD and MCT interventions in older people may improve postural balance by improving vestibular and proprioceptive input, which may translate into older people prolonging single-leg support by shortening the duration of double-leg support, improving the TUG test. In addition, the plantar flexor muscles, which have an important role in the propulsive impulse at the end of the support phase to increase the upward forces that raise and rotate the hip to improve gait, may have increased their capacity for force application following the interventions [61].

### 4.10. 2-min Step Test

For cardiorespiratory fitness measured by the 2-min step test, there were no significant improvements in any groups analyzed in the present study. On the contrary, in the study by Kim, et al. [46] in older females with arterial hypertension, significant improvements in the 2-min step (*p* = 0.003) were presented through an intervention with TKD compared to an inactive CG. These results were similar to what was reported by Jofré-Saldía, et al. [53] in apparently healthy older people with significant improvements in the 2-min step (*p* = 0.000) by MCT compared to an inactive CG. However, Valdés-Badilla, et al. [8], in older females, presented significant improvements only in TKD in the 2-min step test (*p* = 0.0004) compared to MCT. There is evidence that improvements in performance on tests related to lower body muscle strength can improve 2-min step test performance due to increased functionality and efficiency in force production in older people [62]. However, our findings did not report such effects, which could be due to the different baseline values in our participants for the 2-min step and the 30 s chair stand tests compared to the studies above [8,53]. In addition, it may be necessary to develop cardiorespiratory fitness interventions through static and dynamic intermittent walking to improve this capacity in older people.

### 4.11. Limitations and Strengths

The limitations of this study were the following: (i) only including older female; (ii) not analyzing physiological and/or biochemical variables; (iii) the number of the sample was low; (iv) not presenting baseline results for each group, which may influence the results of certain variables; (v) failure to control for external factors such as medication use, daily activity type, and underlying medical condition that could affect the assessment of HRQoL and cognitive status; (vi) not using the same amount of training time for the WE regarding TKD and MCT groups; (vii) the lack of a standardized diet that could affect body fat percentage and fat-free mass; (viii) lack of subsequent follow-up on postural balance and muscle strength, limiting whether improvements are lasting. Among the strengths were the following: (i) the multi-arm study design; (ii) use active and inactive CG; (iii) use certified instructors in TKD and physical activity sciences professionals with experience in training older people; and (iv) applied direct measurements for postural balance and body composition.

### 4.12. Practical Applications

Government agencies such as the Ministry of Health and Sport and local governments can incorporate TKD into their physical activity programs for older people, as it is a safe, low-cost, accessible intervention with good adherence and requires little space, making it a good alternative to conventional interventions. However, interventions must be applied by physical activity and sports professionals who have training in TKD or by TKD instructors certified by the respective federation in physical activity and sports sciences [8,16]. Trained professionals with the necessary knowledge and skills can effectively implement TKD programs. The programs can be conducted in 1 h sessions, 3 days a week, involving non-contact drills, basic stances, and specific technical foundations with the upper (strikes and blocks) and lower (movement, stances, kicks) body, performed individually and in pairs with and without the use of taekwondo impact guards and shields, as well as *poomsae* [1,4]. The moderate–vigorous intensity of the sessions should be monitored via heart rate or perceived exertion [8].

## 5. Conclusions

TKD intervention can improve phonetic fluency of the cognitive status, body pain, and general health dimensions of HRQoL, the 30 s chair stand test, push-up test, sit-up-reach test, and MIHS non-dominant hand of physical fitness, and significantly improve postural balance in EO for the area and mediolateral velocity, and the mediolateral velocity EC in older females. However, TKD equivalently improves HRQoL and physical fitness to MCT, although with better postural balance.

## Figures and Tables

**Table 1:** Time × group interaction in the health-related quality of life dimensions of adapted taekwondo, multicomponent training, walking exercise, and control groups.

	Group	Before		After		Time × Group *p* Value	Time × Group F Value	ηp^2^
		Mean	SD	Mean	SD			
**Physical functioning (%)**	**TKD**	84.6	14.2	91.7	13.2	0.159	1.75	0.081
	MCT	80.0	12.1	91.6	16.5			
	WE	85.3	15.6	77.1	21.7			
	CG	60.8	24.6	64.2	28.7			
**Body pain (%)**	TKD	44.2	19.0	77.3	25.9	**0.041**	3.31	0.122
	MCT	52.2	19.6	81.4	22.8			
	WE	56.6	14.3	63.8	25.2			
	CG	45.0	14.8	63.3	18.3			
**Role limitation-physical (%)**	TKD	91.7	19.5	95.8	14.4	0.924	0.15	0.007
	MCT	93.8	14.4	93.8	19.4			
	WE	97.4	11.5	97.4	11.5			
	CG	91.7	19.5	94.4	19.2			
**Role limitation-emotional (%)**	TKD	95.8	14.4	95.8	14.4	0.542	0.73	0.033
	MCT	93.8	17.1	96.9	12.5			
	WE	90.4	25.6	91.2	24.4			
	CG	86.1	25.5	75.2	22.7			
**Mental health (%)**	TKD	66.3	18.6	66.0	16.0	0.109	0.42	0.089
	MCT	61.0	17.0	67.0	11.5			
	WE	63.6	11.8	60.4	13.5			
	CG	56.3	12.7	70.8	14.4			
**Social functioning (%)**	TKD	65.6	23.3	70.8	24.0	0.945	0.12	0.006
	MCT	62.5	25.4	62.5	18.3			
	WE	69.1	15.8	69.7	24.0			
	CG	47.9	14.9	52.5	13.4			
**Vitality (%)**	TKD	65.8	12.4	69.2	19.5	0.547	0.72	0.038
	MCT	66.9	14.2	58.1	12.9			
	WE	64.7	9.9	59.2	17.1			
	CG	48.3	14.2	45.8	24.8			
**General health (%)**	TKD	54.2	21.4	78.8	20.1	**0.007**	2.27	0.084
	MCT	58.4	13.9	73.1	22.6			
	WE	56.8	17.6	60.5	18.2			
	CG	49.1	16.5	54.1	24.3			

TKD: adapted taekwondo. MCT: multicomponent training. WE: walking exercise. CG: control group. ηp^2^: partial eta square.

**Table 2:** Time × group interaction in the physical fitness assessments of adapted taekwondo, multicomponent training, walking exercise, and control groups.

	Group	Before		After		Time × Group *p* Value	Time × Group F Value	ηp^2^
		Mean	SD	Mean	SD			
**30 s chair stand (rep)**	TKD	17.8	1.9	21.3	2.6	**0.017**	4.25	0.179
	MCT	15.7	2.3	18.9	2.3			
	WE	16.8	3.5	17.0	3.1			
	CG	14.3	1.9	14.8	1.6			
**Arm curl** **(rep)**	TKD	26.1	3.8	29.9	3.8	**<0.001**	8.52	0.299
	MCT	22.8	3.9	28.2	3.4			
	WE	19.9	3.0	20.7	3.9			
	CG	19.8	3.2	19.8	3.2			
**2-min step (rep)**	TKD	112.7	17.3	115.5	5.5	0.737	0.42	0.022
	MCT	93.0	17.5	90.3	16.8			
	WE	88.5	22.5	90.1	24.6			
	CG	78.1	31.1	82.7	19.7			
**Chair sit-and-reach (cm)**	TKD	3.4	2.7	8.3	4.0	**<0.001**	8.56	0.297
	MCT	0.3	4.5	4.2	4.3			
	WE	−1.1	5.5	−0.6	5.7			
	CG	−0.1	3.0	−1.7	3.4			
**Back scratch (cm)**	TKD	−2.9	4.7	−1.1	3.6	0.625	0.59	0.028
	MCT	−6.4	4.7	−7.3	3.9			
	WE	−3.1	4.6	−4.2	7.7			
	CG	−9.0	4.9	−9.0	4.9			
**Timed up-and-go (s)**	TKD	4.9	0.3	3.8	0.6	**0.004**	5.88	0.220
	MCT	5.6	0.8	4.7	0.5			
	WE	5.9	1.2	5.5	1.1			
	CG	5.5	0.7	5.8	1.0			
**MIHS dominant hand (kg)**	TKD	24.3	3.9	25.3	1.2	0.480	0.85	0.044
	MCT	23.1	4.7	24.0	3.2			
	WE	19.5	3.8	19.3	4.5			
	CG	21.3	3.9	21.3	3.9			
**MIHS non-dominant hand (kg)**	TKD	22.9	4.0	25.9	1.6	**0.013**	4.53	0.194
	MCT	21.3	3.6	24.1	2.2			
	WE	17.8	4.5	18.3	5.3			
	CG	19.4	4.1	19.4	4.1			

MIHS: maximal isometric handgrip strength. TKD: adapted taekwondo. MCT: multicomponent training. WE: walking exercise. CG: control group. SD: standard deviation. ηp^2^: partial eta square.

**Table 3:** Time × group interaction in adapted taekwondo’s postural balance (center of pressure), multicomponent training, walking exercise, and control groups.

	Group	Before		After		Time × Group *p* Value	Time × Group F Value	ηp^2^
		Mean	SD	Mean	SD			
**Area EO (m^2^)**	TKD	0.018	0.015	0.007	0.004	**0.007**	5.32	0.222
	MCT	0.016	0.009	0.010	0.008			
	WE	0.014	0.012	0.016	0.010			
	CG	0.007	0.003	0.006	0.003			
**AP velocity EO (m/s)**	TKD	0.453	0.097	0.384	0.029	0.073	2.70	0.128
	MCT	0.507	0.100	0.396	0.130			
	WE	0.531	0.219	0.548	0.213			
	CG	0.376	0.103	0.355	0.068			
**ML velocity EO (m/s)**	TKD	0.403	0.269	0.251	0.024	**0.016**	4.34	0.191
	MCT	0.289	0.094	0.298	0.077			
	WE	0.311	0.093	0.334	0.135			
	CG	0.249	0.031	0.254	0.041			
**Area EC (m^2^)**	TKD	0.016	0.003	0.009	0.004	**0.021**	4.07	0.167
	MCT	0.016	0.003	0.009	0.006			
	WE	0.014	0.013	0.015	0.009			
	CG	0.012	0.016	0.008	0.006			
**AP velocity EC (m/s)**	TKD	0.679	0.147	0.447	0.038	**0.003**	6.50	0.241
	MCT	0.622	0.185	0.443	0.069			
	WE	0.732	0.445	0.700	0.324			
	CG	0.540	0.307	0.568	0.372			
**ML velocity EC (m/s)**	TKD	0.421	0.156	0.247	0.010	**0.003**	6.43	0.213
	MCT	0.376	0.173	0.266	0.017			
	WE	0.385	0.246	0.412	0.258			
	CG	0.280	0.046	0.275	0.074			

AP: anteroposterior. ML: mediolateral. EO: eyes open. EC: eyes closed. TKD: adapted taekwondo. MCT: multicomponent training. WE: walking exercise. CG: control group. SD: standard deviation. ηp^2^: partial eta square.

## Data Availability

The data presented in this study are available on request from the corresponding author due to (specify the reason for the restriction).
